# Metabolic dysregulation in MASLD-associated HCC: diagnostic biomarkers and therapeutic opportunities

**DOI:** 10.3389/fmed.2025.1705723

**Published:** 2025-11-04

**Authors:** Yu Geng, Lanqing Liu, Yongping Sun, Lijuan Guo, Yuanjing Wu, Zhen Jia

**Affiliations:** ^1^Interventional Department, Binhai County People’s Hospital, Yancheng, Jiangsu, China; ^2^Department of Acupuncture and Massage, The First People’s Hospital of Xuzhou, Xuzhou, Jiangsu, China; ^3^Neonatal Medical Center, Children’s Hospital of Nanjing Medical University, Nanjing, Jiangsu, China; ^4^Department of Gynaecology and Obstetrics, Binhai County People’s Hospital, Yancheng, Jiangsu, China; ^5^Department of Laboratory, Haidong Second People’s Hospital, Haidong, Qinghai, China

**Keywords:** MASLD, HCC, metabolic dysregulation, gut microbiota, therapeutic targets

## Abstract

Metabolic dysfunction-associated steatotic liver disease (MASLD), previously referred to as non-alcoholic fatty liver disease (NAFLD), has become the most common chronic liver disease globally, with its incidence rising annually. MASLD is closely linked to metabolic syndrome and can progress from simple steatosis to more severe stages, including non-alcoholic steatohepatitis, fibrosis, cirrhosis, and hepatocellular carcinoma (HCC), affecting 13%–38.2% of cases. Notably, in 40%–50% of patients, this progression occurs without cirrhosis. The dysregulation of glucose and lipid metabolism is a fundamental pathological mechanism in MASLD and its transition to HCC. Key factors include insulin resistance, increased gluconeogenesis, impaired β-oxidation, oxidative stress, and chronic inflammation, all of which contribute to a tumor-promoting hepatic microenvironment. This review provides a comprehensive analysis of the latest research on MASLD-related HCC, emphasizing disturbances in glucose metabolism (such as disrupted hepatic insulin signaling, key enzymes like G6Pase and PK, and miRNAs such as miR-22-3p that induce Warburg effects), lipid imbalances (for example, upregulation of FASN/ACC and downregulation of PPARα targets like CPT1A), and the crosstalk between various pathways (including mTORC1, AMPK/ACC, FXR, and NF-κB/JNK). It also explores metabolic regulators such as DKK3, FGF21, and O-GlcNAcylation, and examines the role of the gut microbiota in modulating short-chain fatty acids, bile acids, and NLRP3 inflammasome activation in disease progression. By integrating the latest advancements in basic and clinical research, this article presents a solid theoretical framework for early diagnosis, risk assessment, biomarker development, and precision therapies. It also highlights promising therapeutic targets, including PPARα agonists, mTOR inhibitors, FGF21 analogs, and microbiota interventions, while proposing future directions in multi-omics and personalized treatment strategies.

## 1 Introduction

Metabolic dysfunction-associated steatotic liver disease (MASLD), previously known as non-alcoholic fatty liver disease (NAFLD), represents a paradigm shift in nomenclature, as endorsed by the 2023 AASLD/EASL consensus (1). Unlike traditional NAFLD, which emphasized the exclusion of alcohol as an etiology, MASLD positively defines the condition by the presence of hepatic steatosis (≥ 5%) coupled with at least one cardiometabolic risk factor (e.g., obesity, type 2 diabetes, dyslipidemia), underscoring metabolic dysfunction as the primary driver (2). Pathophysiologically, this redefinition highlights insulin resistance and lipotoxicity as central mechanisms, distinguishing MASLD from broader NAFLD spectra that included non-metabolic idiopathic steatosis. MASLD is now recognized as a major contributor to cirrhosis and hepatocellular carcinoma (HCC). The progression of this condition unfolds in several distinct stages, beginning with simple hepatic steatosis (NAFL), which can advance to non-alcoholic steatohepatitis (NASH) and may subsequently progress to liver fibrosis, cirrhosis, or eventually HCC (3). The Global Burden of Disease study has identified MASLD as a significant and rapidly growing risk factor for liver cancer, with notably higher incidence rates observed in younger individuals and males (4). In Western countries, MASLD has become the leading risk factor for hepatocellular carcinoma (5).

The primary mechanisms underlying MASLD are closely linked to the dysregulation of glucose and lipid metabolism, encompassing insulin resistance, lipid accumulation, inflammatory responses, oxidative stress, and other related pathological processes (6, 7). Research indicates that excessive consumption of high-fat and high-sugar diets is a major factor contributing to the development of MASLD. These dietary patterns promote abnormal fat accumulation in the liver, which in turn triggers the onset of chronic liver disease (8, 9). Moreover, the progression of MASLD is multifaceted, involving the interplay of various pathways, including metabolic dysregulation, liver inflammation, cell apoptosis, and fibrosis (10, 11). The pathological features of MASLD-related HCC are notably complex, presenting clinical and molecular characteristics that differ from those of conventional cirrhotic liver cancer. In patients with MASLD, around 40%–50% of HCC cases develop in the absence of apparent cirrhosis (12). This phenomenon presents considerable challenges to traditional HCC screening methods, which rely on the presence of liver cirrhosis. Research into the underlying mechanisms of MASLD-related HCC has revealed that these processes are closely linked to prolonged metabolic stress, including insulin resistance, hyperglycemia, lipotoxicity, oxidative stress, and chronic inflammation. Together, these factors create a hepatic microenvironment conducive to the development of liver cancer.

Currently, effective treatment options for MASLD and its associated liver cancer are limited. Traditional approaches primarily focus on lifestyle modifications, including weight loss, dietary improvements, and increased physical activity, to help reduce the risk of disease development (13, 14). For patients already diagnosed with MASLD, regular monitoring and early screening are essential. Moreover, for individuals with NASH or those at high risk, personalized treatment strategies—such as pharmacological interventions to address insulin resistance or non-invasive therapies to reduce lipotoxicity—are critical. A thorough understanding of the regulatory mechanisms governing glucose and lipid metabolism is therefore key to developing new therapeutic approaches. Researchers are increasingly investigating potential therapeutic targets, including AMP-activated protein kinase (AMPK), liver X receptor (LXR), and farnesoid X receptor (FXR), all of which play pivotal roles in regulating glucose and lipid metabolism as well as inflammatory responses (15, 16). For instance, the activation of AMPK is believed to reduce hepatic lipid accumulation and insulin resistance, thereby helping to slow the progression of MASLD (17). Additionally, modulating hepatic metabolic signaling pathways, such as enhancing metabolic parameters with sodium-glucose cotransporter type 2 (SGLT2) inhibitors, offers a promising new approach for the treatment of MASLD (18).

In conclusion, as our understanding of the connection between MASLD and HCC advances, the medical community is actively seeking more effective treatment strategies to prevent liver cancer and slow disease progression. Future research will focus on elucidating the causal relationships between these conditions, developing novel biomarkers and diagnostic tools, and creating more targeted treatment approaches. Additionally, raising public health awareness and implementing supportive public health policies will be crucial to addressing this issue. Only through the integrated application of multidisciplinary knowledge and techniques can we more effectively tackle this global health challenge. This paper will focus on the role of glucose and lipid metabolism in MASLD-related liver cancer, systematically reviewing the latest research to provide a theoretical foundation for understanding MASLD and its progression, with the goal of offering valuable insights for future clinical treatment strategies.

## 2 Dysregulation of glucose and lipid metabolism in MASLD-related hepatocellular carcinoma

### 2.1 Central role of glucose and lipid metabolism

The disruption of glucose and lipid metabolism is a key driver in the development of MASLD and its progression to HCC. This disruption encompasses several processes, including insulin resistance, increased gluconeogenesis, elevated fatty acid synthesis, impaired β-oxidation, oxidative stress, and inflammatory responses. These metabolic abnormalities are interconnected and work together to promote hepatic fat accumulation, inflammation, fibrosis, and the formation of a carcinogenic microenvironment, ultimately facilitating the progression from MASLD to NASH and HCC.

Abnormal glucose metabolism in MASLD is mainly characterized by insulin resistance, increased gluconeogenesis, and impaired glucose utilization. Patients with MASLD typically display marked insulin resistance, which disrupts hepatic glucose metabolism and triggers systemic metabolic disturbances. This, in turn, exacerbates hepatic fat accumulation and inflammation (19, 20). Increased gluconeogenesis leads to continuous glucose release from the liver, worsening hyperglycemia and, in turn, promoting fat accumulation, thereby establishing a vicious cycle. Additionally, disruptions in vitamin A metabolism also impact glucose metabolism. Vitamin A modulates glucose metabolism through retinoic acid-mediated transcriptional networks (21). In MASLD, the PNPLA3-I148M variant is linked to reduced serum retinol levels and elevated hepatic retinyl esters, indicating that an imbalance in vitamin A metabolism may contribute to disease progression.

Abnormal lipid metabolism in MASLD is characterized by increased hepatic fatty acid synthesis, impaired β-oxidation, and lipid accumulation. Under normal conditions, the liver converts fatty acids into energy through β-oxidation. However, in MASLD, this process is inhibited, resulting in fatty acid buildup and the development of hepatocellular steatosis (22). As MASLD progresses to NASH and liver fibrosis, fatty acid synthesis intensifies, further stressing the liver and promoting inflammation and fibrosis. These processes create a conducive environment for the development of liver cancer (23). In a diet-induced MASLD mouse model, increased hepatic triglyceride and cholesterol levels, along with heightened insulin resistance and inflammation, were closely linked to the development of HCC (24).

Certain bioactive molecules play essential regulatory roles in glucose and lipid metabolism, as well as in liver cancer development. For example, Dickkopf-3 (DKK3) is significantly downregulated in the livers of MASLD patients and obese mice. Hepatocyte-specific overexpression of DKK3 improves insulin sensitivity, glucose tolerance, lipid metabolism, and suppresses inflammation, while DKK3 deficiency exacerbates pathological changes (25). Mechanistically, DKK3 inhibits the activation of the P38/JNK pathway by binding to apoptosis signal-regulating kinase 1 (ASK1) (26).

Disruption of glucose and lipid metabolism is a key factor in the pathogenesis of MASLD and its associated liver cancer. Through mechanisms such as insulin resistance, fat accumulation, oxidative stress, and inflammation, these metabolic abnormalities collectively contribute to liver damage and the creation of a carcinogenic microenvironment. Imbalances in vitamin A metabolism and alterations in the gut microbiome further aggravate this process. Interventions aimed at regulating glucose and lipid metabolism, including targeting key molecules like DKK3, may offer promising new therapeutic strategies for MASLD and related liver cancer.

### 2.2 The link between glucose and lipid metabolism dysregulation and hepatocellular carcinoma development

Abnormal glucose and lipid metabolism plays a critical role in the development of HCC, primarily by promoting cell proliferation, inhibiting apoptosis, and inducing genetic mutations. MASLD is recognized as a significant risk factor for HCC, with studies showing that 13%–38.2% of MASLD patients will develop HCC, especially in cases where the disease progresses to NASH (27). As MASLD progresses, dysregulation of lipid metabolism results in abnormal fat accumulation in the liver, which triggers inflammatory responses and apoptosis in cells, thereby promoting the proliferation and survival of cancer cells. Specifically, metabolic reprogramming allows tumor cells to thrive in hypoxic and nutrient-poor environments, using fatty acids as an energy source to fuel their rapid proliferation and growth (28, 29).

Dysregulation of glucose and lipid metabolism induces oxidative stress and inflammatory responses, which are critical in the progression from MASLD to liver cancer. In the livers of MASLD patients, oxidative stress markers are elevated, correlating with lipid peroxidation and cellular damage (30, 31). Oxidative stress not only damages hepatocytes but also accelerates liver tissue fibrosis. Chronic inflammation, driven by fat accumulation, exacerbates hepatocyte injury and contributes to the formation of a tumor microenvironment, acting as a catalyst for both fibrosis and HCC (32). Additionally, alterations in the gut microbiota, through the modulation of short-chain fatty acid production, impact liver inflammation and immune responses, thereby contributing to the development of liver cancer.

Furthermore, studies have shown that lipid metabolism disorders associated with MASLD are linked to the occurrence of genetic mutations. For instance, the activation of transcription factors such as E2F1 and E2F2 is closely associated with HCC development. These factors regulate fatty acid oxidation and synthesis, which in turn affect the metabolic state of hepatocytes, promoting the progression of HCC (6). Abnormal glucose and lipid metabolism not only impacts hepatocyte proliferation and apoptosis but also, by altering the liver’s microenvironment, promotes fibrosis, ultimately contributing to the development of HCC (33).

In terms of metabolic reprogramming, tumor cells increase lipid synthesis and suppress fatty acid oxidation to acquire more energy and biosynthetic materials. This metabolic shift enables tumor cells to establish a microenvironment within the liver that supports their growth, thereby further accelerating the onset and progression of liver cancer (34). Understanding the role of glucose and lipid metabolism in HCC development is crucial, as it not only enhances our comprehension of the pathogenesis of liver cancer but also identifies potential targets for new therapeutic strategies. By intervening in these metabolic pathways, novel approaches for the prevention and treatment of liver cancer may be uncovered.

## 3 The Role of glucose metabolism in MASLD-related hepatocellular carcinoma

### 3.1 Abnormal hepatic insulin signaling pathway

In MASLD and its associated HCC, abnormalities in the hepatic insulin signaling pathway play a crucial role in the dysregulation of glucose metabolism. Disruption of this pathway not only increases gluconeogenesis but also impairs glucose utilization (20, 35). Under normal physiological conditions, insulin activates its receptor to promote glucose uptake and storage in the liver, stimulates glycogen synthesis, and inhibits gluconeogenesis. However, in MASLD patients, the onset of insulin resistance diminishes the liver’s ability to respond to insulin, resulting in enhanced gluconeogenesis and elevated blood glucose levels (36).

Abnormalities in the insulin signaling pathway also disrupt lipid metabolism, promoting lipid synthesis and creating a harmful metabolic cycle. Studies have shown that in the state of insulin resistance, the liver continues to promote lipid synthesis while failing to adequately suppress gluconeogenesis, resulting in abnormal elevations in both blood glucose and blood lipids (37, 38). This vicious cycle not only worsens MASLD but may also accelerate its progression to more severe stages, such as NASH and liver cancer.

In-depth research into the hepatic insulin signaling pathway has revealed several molecular pathways involved in this process. One such pathway involves AMPK, a key metabolic regulator that plays a critical role in insulin signaling. Activation of AMPK enhances insulin sensitivity, inhibits hepatic lipid synthesis, and promotes fatty acid oxidation, thereby helping to mitigate the progression of MASLD (39). Additionally, vascular endothelial growth factor B (VEGFB) has been shown to improve insulin resistance and lipid metabolism by modulating the PI3K/AKT pathway (40).

Abnormalities in the hepatic insulin signaling pathway not only disrupt glucose and lipid metabolism but are also closely linked to chronic low-grade inflammation, oxidative stress, and other mechanisms that drive the onset and progression of MASLD. As a result, targeting the insulin signaling pathway could represent a key therapeutic strategy for MASLD and its associated liver cancer. Enhancing the function of this pathway may help mitigate the progression of MASLD and reduce the risk of liver cancer development.

### 3.2 Regulation of key enzymes and transporters

In the study of MASLD-related HCC, the regulation of key enzymes and transporters is crucial for maintaining the balance of glucose and lipid metabolism. Alterations in the activity of key enzymes, such as hepatic glucose-6-phosphatase and pyruvate kinase, can directly impact hepatic glucose metabolism. For instance, glucose-6-phosphatase (G6Pase) plays a vital role in converting glucose-6-phosphate to glucose in the liver, a process central to gluconeogenesis. Research indicates that in MASLD, the expression of G6Pase may be upregulated, leading to increased hepatic gluconeogenesis and exacerbating hepatic fat accumulation (41, 42). In contrast, pyruvate kinase (PK), a key enzyme in glycolysis, when less active, drives a shift in metabolism toward fat synthesis, playing a crucial role in the pathological progression of MASLD. Furthermore, studies have shown that inhibiting PK activity can reduce hepatic fat accumulation, highlighting its potential therapeutic value in MASLD and liver cancer (43).

On the other hand, intestinal gluconeogenesis (IGN) plays a significant role in regulating hepatic glucose metabolism. IGN refers to the glucose produced in the intestines following the absorption of specific nutrients, facilitated by an intestinal-brain-liver neural signaling mechanism. Studies have shown that IGN can effectively prevent hepatic steatosis and mitigate the onset of MASLD by reducing hepatic fat synthesis and lipid influx. Specifically, transgenic mice with enhanced IGN exhibit a marked reduction in hepatic fat accumulation when fed high-calorie diets, a phenomenon closely linked to decreased hepatic inflammation and fibrosis (41, 44). Furthermore, the upregulation of IGN is closely linked to the balance between hepatic gluconeogenesis and lipogenesis, offering new potential targets for the treatment of MASLD.

In conclusion, key enzymes (such as G6Pase and PK) and transporters are central to the regulation of glucose and lipid metabolism in MASLD, while intestinal gluconeogenesis presents a novel approach for regulating hepatic glucose metabolism. These findings offer a theoretical foundation for developing MASLD treatment strategies. Future research could focus on therapeutic interventions targeting these key enzymes and transporters to restore metabolic balance, thereby slowing the progression of MASLD.

### 3.3 Mechanisms of miRNA regulation of glucose metabolism

In the liver, miR-22-3p is a key regulator of glucose metabolism and plays a crucial role in the development of MASLD and liver cancer. Research has shown that miR-22-3p is among the most abundant microRNAs (miRNAs) in the liver, with alterations in its expression closely linked to hepatic steatosis, insulin resistance, and cancer progression (45). Downregulation of miR-22-3p is frequently observed in liver cancer, suggesting its potential involvement in the pathophysiology of liver-related metabolic disorders. However, the precise role and mechanisms of miR-22-3p in the context of obesity and MASLD (metabolic-associated steatotic liver disease) remain unclear, warranting further investigation to elucidate its effects. In miR-22 knockout (miR-22KO) mice subjected to a high-fat diet to induce obesity, no significant symptoms were observed under normal conditions. However, under the high-fat diet challenge, miR-22KO mice exhibited marked glucose intolerance, fat accumulation, hepatomegaly, and hepatic steatosis. Further analysis revealed an upregulation of glycolytic and lipid uptake enzymes in the liver, highlighting the critical role of miR-22 in regulating hepatic metabolism.

Furthermore, the expression of miR-22-3p in Huh7 liver cancer cells was found to be inversely related to its expression in non-tumor tissues, displaying a distinct “Warburg effect,” characterized by increased glycolysis and suppressed mitochondrial respiration (46, 47). Specifically, this “Warburg effect” is achieved because miR-22-3p directly targets and suppresses the expression of key enzymes involved in the mitochondrial tricarboxylic acid (TCA) cycle and oxidative phosphorylation. This metabolic shift toward aerobic glycolysis, even in the presence of oxygen, provides rapidly proliferating cancer cells with not only ATP but also essential biosynthetic precursors for nucleotide and lipid synthesis, thereby fueling tumor growth and progression. This observation suggests that miR-22-3p may exert distinct regulatory effects under different physiological and pathological conditions, thereby influencing both hepatic metabolism and tumor development. These findings highlight the potential of miR-22-3p as a therapeutic target, especially in the treatment of obesity-associated MASLD and liver cancer, where altering its expression or function could offer novel therapeutic approaches for patients.

In conclusion, miR-22-3p plays a key role in regulating hepatic glucose metabolism, and its loss may contribute to the progression of obesity and MASLD, ultimately influencing the development of liver cancer. A deeper investigation into this mechanism will improve our understanding of the pathological processes underlying MASLD and hepatocellular carcinoma, offering new targets and strategies for therapeutic intervention. It is important to note that the regulatory functions of miRNAs such as miR-22-3p are not isolated; rather, they are integral components of the broader metabolic signaling network. They often serve as critical nodes that interact with other major pathways discussed in this reviewte to the progI3K/AKT and AMPK signaling cascades They often serve as critical nodes that interact wit(19). Understanding this interplay is essential for developing targeted therapies capable of restoring metabolic homeostasis.

## 4 Lipid metabolism abnormalities and their role in MASLD-related hepatocellular carcinoma

### 4.1 Imbalance between fatty acid synthesis and oxidation

The imbalance between fatty acid synthesis and oxidation is a critical factor in the development of MASLD and its progression to associated HCC. This condition is often characterized by altered expression of key enzymes, including fatty acid synthase (FASN) and acetyl-CoA carboxylase (ACC). The upregulation of these enzymes enhances fatty acid synthesis in the liver, leading to excessive fatty acid accumulation. This accumulation can disrupt hepatocyte function and trigger inflammation, thereby accelerating the pathological progression of the disease (27, 48). Studies have demonstrated that in MASLD patients, the expression levels of FASN and ACC are markedly elevated, suggesting that the activation of the fatty acid synthesis pathway is closely linked to the pathophysiological changes associated with MASLD (49, 50). Moreover, the imbalance in fatty acid synthesis is closely linked to insulin resistance, inflammation, and liver fibrosis, all of which collectively contribute to the progression toward HCC (19).

At the same time, impaired β-oxidation of fatty acids plays a crucial role in the progression of MASLD. Under normal conditions, fatty acids undergo β-oxidation in the mitochondria, generating energy for cellular functions. However, in MASLD patients, this process is frequently disrupted, resulting in the accumulation of fatty acids within hepatocytes. This accumulation further exacerbates hepatocyte damage and promotes apoptosis (28, 51). For instance, transcription factors like E2F1 and E2F2 have been implicated in the metabolic reprogramming of fatty acids in MASLD-related HCC. Their elevated expression is inversely associated with the downregulation of enzymes involved in fatty acid β-oxidation, such as carnitine palmitoyltransferase 2 (CPT2), highlighting their significant role in fatty acid metabolism (22). This metabolic reprogramming not only worsens the fatty accumulation in the liver but also facilitates the malignant transformation of hepatocytes. In addition, lipotoxicity arises from the imbalance in fatty acid synthesis/oxidation, where excess saturated free fatty acids induce hepatocyte damage via multiple interconnected pathways. Overloaded mitochondria generate ROS, triggering ER stress and unfolded protein response (UPR), which activates JNK-mediated apoptosis (52). Concurrently, diacylglycerol accumulation inhibits insulin signaling through PKC translocation, exacerbating hyperglycemia and ceramide synthesis; ceramides, in turn, activate NLRP3 inflammasome, amplifying inflammation and fibrosis. These lipotoxic cascades foster a protumorigenic microenvironment, promoting HCC via genomic instability and immune evasion. Targeting lipotoxicity (e.g., via SCD1 inhibition) thus offers dual benefits in halting MASLD progression and HCC oncogenesis.

The imbalance between fatty acid synthesis and oxidation represents not only a key pathological change in the progression of MASLD but also serves as a biological foundation for its progression to HCC. Researchers are increasingly focused on developing interventions that target these metabolic pathways, aiming to offer new therapeutic strategies for MASLD and its associated complications. By gaining a deeper understanding of fatty acid metabolism in MASLD development, more effective preventive and therapeutic measures can be devised, ultimately improving patient outcomes and reducing the incidence of HCC.

### 4.2 Regulatory role of PPARα and its target genes in lipid metabolism

Peroxisome proliferator-activated receptor alpha (PPARα) is a key regulator of lipid metabolism, primarily influencing fatty acid oxidation and inflammatory responses. It is highly expressed in the liver, where it plays a crucial role in the uptake, esterification, and transport of fatty acids, as well as regulating the expression of genes involved in lipoprotein metabolism (53). Studies have demonstrated that PPARα enhances fatty acid β-oxidation by activating target genes, such as carnitine palmitoyltransferase 1A (CPT1A) and peroxisomal acyl-CoA oxidase 1 (ACOX1), thereby helping to maintain lipid homeostasis (54, 55). Additionally, PPARα plays a role in inhibiting hepatic inflammation, reducing liver fibrosis, and preventing hepatic steatosis, thereby mitigating the progression of MASLD and NASH (56). The role of PPARα in MASLD and NASH is particularly significant. Its agonists, such as fenofibrate, have been shown to notably improve lipid metabolism and reduce hepatic inflammation (57).

Clinical trials have highlighted the potential of PPARα agonists in reducing lipid levels and improving liver function, particularly in the treatment of MASLD and NASH (58, 59). However, the expression and activity of PPARα decrease as MASLD and NASH progress, likely due to impaired fatty acid metabolism and heightened hepatic inflammation (60). The use of PPARα agonists in treating MASLD and NASH has spurred extensive research. Several PPARα agonists are currently undergoing clinical trials, demonstrating effectiveness in reducing liver fat accumulation and improving liver function. For instance, some novel PPARα agonists notably enhance the expression of genes related to fatty acid oxidation in the liver, reduce hepatic inflammation, and improve the metabolic status of patients (61). These studies provide a crucial theoretical basis for considering PPARα as a potential therapeutic target for MASLD and NASH. Future research may further clarify its role in other metabolic disorders. Recent clinical data further support the applicability of PPARα agonists. For example, lanifibranor, a pan-PPAR agonist, has advanced to Phase 3 trials (as of 2025), showing significant reductions in liver fat (up to 45%), fibrosis improvement, and enhanced insulin sensitivity in MASH patients (62). However, challenges include mild gastrointestinal side effects and the need for long-term monitoring of efficacy in HCC prevention. Real-world evidence from post-marketing studies of fenofibrate indicates sustained lipid-lowering effects but variable fibrosis resolution, emphasizing the importance of combination therapies for optimal outcomes (63).

In conclusion, PPARα plays a key regulatory role in lipid metabolism, and its agonists hold significant therapeutic potential for MASLD and NASH. Future research is anticipated to offer new insights and evidence to support the development of more effective treatment strategies.

### 4.3 Crosstalk between glucose and lipid metabolism and the critical signaling pathway

#### 4.3.1 mTOR signaling pathway

mTORC1 (mechanistic target of rapamycin complex 1), a specific complex of mTOR, plays a pivotal role in regulating glucose and lipid metabolism. It governs critical processes such as fatty acid synthesis, glycolysis, and amino acid metabolism. By modulating energy balance through pathways like the AMPK signaling pathway, mTORC1 influences hepatocyte metabolism. In MASLD and liver cancer, the activation of mTORC1 is linked to increased lipid accumulation and inflammation. Elevated fatty acid levels activate mTORC1, which in turn promotes lipid synthesis and glucose metabolism, driving the progression of MASLD (64). In liver cancer, mTORC1 activation promotes cell proliferation, inhibits apoptosis, and triggers metabolic reprogramming, thereby supporting tumorigenesis (65). mTORC1 regulates key downstream effectors, such as S6K1 and 4E-BP1, which promote hepatocyte growth and support tumor cell survival (66).

Excessive activation of mTORC1 profoundly alters cellular metabolism, driving hepatocyte proliferation and facilitating the development of MASLD-related liver cancer. In MASLD, elevated fatty acid levels activate mTORC1, enhancing lipid synthesis and cell proliferation while suppressing apoptosis, thereby accelerating the progression of liver cancer (67). mTORC1 activation also upregulates genes such as sterol regulatory element binding factor 1 (SREBF1) and fatty acid synthase (FASN), thereby promoting lipid accumulation and hepatocyte growth (68). argeting mTORC1 with inhibitors such as rapamycin has demonstrated potential in reducing liver cancer cell proliferation and survival, particularly in models of MASLD-associated liver cancer (69). This suggests that inhibiting mTORC1 could provide innovative therapeutic approaches for treating both MASLD and liver cancer.

#### 4.3.2 AMPK/ACC signaling pathway

The AMPK/ACC pathway has garnered significant attention in studies of HCC associated with MASLD. As a key regulator of cellular energy balance, AMPK plays a critical role in liver cancer development by influencing lipid metabolism, glycolysis, and the cell cycle (70). Activation of AMPK inhibits acetyl-CoA carboxylase (ACC), thereby reducing fatty acid synthesis and promoting fatty acid oxidation, which offers protective effects in MASLD (71). Increased expression of sebum protein P (SeP) in MASLD promotes lipid accumulation via the AMPK/ACC pathway. Inhibiting SeP can reduce triglyceride accumulation, presenting a potential novel target for diagnosis and treatment (72). Additionally, 5-aminobutyric acid (5-ALA) activates AMPK, highlighting its essential role in regulating lipid metabolism in a high-fat diet-induced mouse model (73).

Dysregulated lipid metabolism is a key factor in the development of liver cancer. FASN has been shown to promote colorectal cancer cell proliferation and metastasis through the AMPK/mTOR pathway, a mechanism that may also be relevant to liver cancer (74). Furthermore, CD147 reprograms fatty acid metabolism in liver cancer cells through the Akt/mTOR/SREBP1c and P38/PPARα pathways, further underscoring the critical role of lipid metabolism in liver cancer (75). In conclusion, the AMPK/ACC pathway plays a crucial role in regulating lipid metabolism and provides new insights into the diagnosis and treatment of MASLD-related liver cancer. Further investigation of this pathway and its molecular mechanisms could pave the way for more effective strategies in preventing and treating MASLD and associated liver cancers.

#### 4.3.3 FXR signaling pathway

Farnesoid X receptor (FXR), a nuclear receptor that primarily regulates bile acid metabolism and plays a key role in lipid and glucose metabolism, is essential in the pathophysiology of MASLD-related liver cancer. Dysregulation of FXR in both MASLD and HCC may contribute to disease progression and worsening (76). FXR agonists, such as Obeticholic Acid (OCA), have demonstrated therapeutic potential in treating NASH by improving fibrosis and potentially delaying the progression to cirrhosis. However, side effects, including alterations in atherosclerotic lipid profiles, may necessitate adjunctive therapy with statins (77, 78).

The role of FXR in the gut-liver axis has been extensively studied, with intestinal microbiota dysbiosis identified as a factor in the progression of both MASLD and HCC. FXR regulates bile acid metabolism and maintains gut microbiota balance, influencing intestinal permeability and immune activation, which may, in turn, affect MASLD progression (79). Combined regulation of FXR with other targets, such as soluble epoxide hydrolase (sEH), has shown enhanced therapeutic effects in NASH, particularly in terms of anti-inflammatory and anti-fibrotic outcomes (80). In conclusion, the regulatory mechanisms of FXR and its involvement in the gut-liver axis offer significant clinical and research potential for the prevention and treatment of MASLD and HCC, particularly through multi-target therapeutic approaches.

#### 4.3.4 NF-κB/JNK signaling pathway

The NF-κB pathway plays a pivotal role in liver cancer progression, with its activation associated with an unfavorable prognosis and stem cell-like traits (81). Dysregulated NF-κB activation is linked to a range of inflammatory diseases and cancers, positioning it as a promising target for therapeutic intervention (82). In MASLD-associated HCC, NF-κB activation drives tumor progression and is closely linked to hepatic fibrosis. Natural compounds that modulate this pathway have demonstrated potential in reducing fibrosis and in the development of novel anti-fibrotic therapies (83). Furthermore, the non-classical NF-κB pathway plays a critical role in the regulation of liver diseases, with small molecule inhibitors emerging as promising candidates for managing liver injury (84).

The JNK pathway is also essential in HCC, impacting cell death, proliferation, and carcinogenesis. Studies indicate a cross-regulation between the JNK and NF-κB pathways, which may affect tumor progression by modulating cellular survival and apoptosis (85). JNK activation is associated with oxidative stress and mitochondrial dysfunction, driving hepatocyte apoptosis in cancer cells (86). In the treatment of MASLD-related liver cancer, targeting the NF-κB and JNK pathways may provide therapeutic benefits. Certain natural products and compounds have shown potential in inhibiting liver cancer cell proliferation and migration by suppressing these pathways (87, 88). Moreover, microRNAs like miR-26b enhance chemotherapy sensitivity by inhibiting NF-κB, offering promising new therapeutic strategies (89). In conclusion, the JNK and NF-κB pathways are crucial in MASLD-related liver cancer, representing promising targets for the development of novel diagnostic and therapeutic approaches.

## 5 Metabolic regulatory factors and their role in MASLD-Related hepatocellular carcinoma

### 5.1 Fibroblast growth factor 21

Fibroblast growth factor 21 (FGF21) is a hormone-like protein predominantly produced in the liver and adipose tissue, playing a key role in regulating glucose and lipid metabolism. Recent studies have shown that FGF21 enhances insulin sensitivity and reduces hepatic fat accumulation, offering protective effects against MASLD and its progression to HCC (90). FGF21 regulates lipid metabolism by inhibiting lipogenesis and enhancing insulin sensitivity in the liver, thereby improving its lipid profile (91). During MASLD progression, FGF21 expression levels are often linked to disease severity. A deficiency in FGF21 worsens conditions that promote liver tumorigenesis, thereby increasing the risk of HCC (92).

Clinical research has established a connection between genetic variations in FGF21 and patients’ dietary patterns as well as addictive behaviors, including smoking and alcohol consumption. This highlights the gene’s significant role in metabolic disorders (90). Moreover, FGF21 analogs, including Pegbelfermin, are being explored as potential treatments for MASLD and NASH. These compounds have shown promise in enhancing liver metabolic health, with clinical trials demonstrating favorable safety and efficacy profiles (93). The role of FGF21 as a biomarker in MASLD and HCC is gaining increasing recognition. Elevated serum levels of FGF21 are now regarded as a key indicator of MASLD, reflecting metabolic disturbances that occur throughout the disease progression (94). Studies have also shown that the upregulation of FGF21 is linked to the progression of various cancers, including breast cancer. This suggests that FGF21 may contribute to tumor growth within the tumor microenvironment (95). Thus, FGF21 not only holds promise as a potential biomarker for MASLD but may also serve as a therapeutic target for cancers like HCC. Clinical applicability of FGF21 analogs has been demonstrated in recent Phase 2 trials (2023-2025). Pegozafermin reduced liver fat by 27%–42% and improved fibrosis in MASH patients, with good tolerability but potential injection-site reactions (96). Efruxifermin showed efficacy in compensated cirrhosis due to MASH, resolving steatohepatitis in 41% of cases, though long-term HCC prevention data are limited (97). Challenges include variable patient responses and the need for combination with other agents for enhanced efficacy.

While research on FGF21 continues to evolve, current findings offer solid theoretical support for its potential use in treating MASLD and HCC. Future studies should prioritize the clinical application of FGF21 and its analogs, investigating their mechanisms across various stages of MASLD and HCC to inform the development of more effective therapeutic strategies.

### 5.2 O-GlcNAc modification and its metabolic regulatory functions

O-GlcNAc modification is a crucial post-translational modification that regulates cellular metabolic processes by detecting changes in nutrient levels through UDP-GlcNAc, which is derived from the hexosamine biosynthesis pathway (HBP). This modification is integral to the development of various diseases, particularly metabolic disorders such as diabetes, cancer, and neurodegenerative diseases, where its dysregulation is recognized as a key driver of disease progression (98, 99). In response to acute stress, O-GlcNAc modification is rapidly upregulated, boosting cellular stress resistance and promoting survival (100). Studies have demonstrated that O-GlcNAcylation regulates enzymes involved in lipid metabolism, such as FASN, thereby supporting tumor growth and survival (101, 102).

In MASLD, dysregulated O-GlcNAcylation is linked to hepatic lipid accumulation, insulin resistance, and the progression of liver cancer (103, 104). O-GlcNAcylation also modulates insulin signaling, impacting insulin secretion and β-cell function (105). O-GlcNAc transferase (OGT), the key enzyme regulating this modification, plays a pivotal role in the progression of MASLD, particularly in modulating mitochondrial function and oxidative stress (106). Inhibiting OGT has demonstrated potential in enhancing mitochondrial function, reducing lipid accumulation, and alleviating inflammation, positioning OGT as a promising therapeutic target for MASLD (103). In conclusion, O-GlcNAc modification, regulated by OGT, plays a crucial role in metabolic diseases like MASLD. Targeting OGT may provide innovative therapeutic approaches for these conditions.

## 6 Gut microbiota and glucose-lipid metabolism interactions inmasld-related hepatocellular carcinoma

### 6.1 Impact of gut microbiota dysbiosis on liver metabolism

Alterations in the composition of the gut microbiota are closely associated with liver glucose and lipid metabolism, as well as inflammatory responses. Recent studies have underscored the crucial role of the gut microbiota in the pathogenesis of MASLD, particularly in the regulation of hepatic lipid metabolism and inflammation. Dysbiosis, or microbial imbalance, triggers inflammation, which is recognized as a key factor in the progression of MASLD. When the intestinal barrier is compromised, harmful substances such as bacterial endotoxins can enter the bloodstream, leading to hepatic inflammation and metabolic disturbances (107). Studies indicates that the gut microbiota, through its metabolic byproducts such as short-chain fatty acids and bile acids, plays a key role in regulating hepatic lipid metabolism and immune responses. This, in turn, influences hepatocyte function, impacting lipid accumulation and inflammatory processes (108).

Alterations in specific gut microbiota, such as an elevated abundance of *Desulfovibrio*, have been associated with the progression of MASLD. *Desulfovibrio* is a bacterium known for metabolizing sulfur compounds, and its metabolic byproducts may modulate liver lipid metabolism and inflammatory processes, thereby contributing to the development of MASLD (109). As a prominent sulfate-reducing bacterium, an overgrowth of Desulfovibrio leads to excessive production of hydrogen sulfide (H2S). At high concentrations, H2S can exert cytotoxic effects, impairing the integrity of the intestinal epithelial barrier and increasing its permeability. This ‘leaky gut’ phenomenon facilitates the translocation of bacterial endotoxins into the portal circulation, directly fueling hepatic inflammation. Furthermore, dysbiosis is closely linked to other components of metabolic syndrome, including insulin resistance, all of which contribute to abnormal hepatic fat accumulation and hepatocyte damage (110). In the management of MASLD, modulation of the gut microbiota has garnered significant interest. Strategies such as probiotics, prebiotics, and fecal microbiota transplantation are recognized as effective in enhancing gut microbiota balance and mitigating liver inflammation and fat accumulation (111). These studies offer valuable insights into the prevention and treatment of MASLD, highlighting the essential role of the gut microbiota in liver metabolism.

In conclusion, dysbiosis of the gut microbiota affects liver metabolic function and plays a critical role in the progression of MASLD by modulating inflammatory responses. Future research should focus on uncovering the underlying mechanisms of gut microbiota regulation and its potential therapeutic applications in preventing and treating MASLD, providing new strategies for clinical management.

### 6.2 Gut-liver axis mechanisms and regulation of metabolites

The gut-liver axis describes the bidirectional communication between the gut and liver, where metabolites produced by the gut microbiota regulate liver metabolism and immune function. Recent studies highlight the significant roles of metabolites, including short-chain fatty acids (SCFAs) and bile acids, in this process. SCFAs, produced through the fermentation of dietary fibers by gut microbiota, not only supply energy to intestinal cells but also impact liver health by modulating lipid metabolism and inflammatory responses (112). SCFAs can activate GLP-1 (glucagon-like peptide-1) receptors, which stimulates insulin secretion and enhances insulin sensitivity, thereby helping to reduce the risk of hepatic fat accumulation (113). Similarly, bile acids, as key signaling molecules between the liver and gut, regulate lipid metabolism by influencing hepatic metabolic functions through feedback mechanisms (114). Dysregulation of bile acids is strongly linked to the development of MASLD, with research indicating that impaired bile acid metabolism in the liver may worsen fatty liver disease.

Additionally, the integrity of the gut barrier is essential for the proper functioning of the gut-liver axis. When the intestinal barrier is compromised, endotoxins like gut-derived lipopolysaccharides (LPS) can enter the bloodstream, triggering chronic inflammation in the liver and accelerating the progression of fatty liver disease (115). Studies have demonstrated that when the gut barrier is compromised, bacterial components such as LPS can reach the liver, where they activate immune cells and trigger excessive inflammatory responses (116, 117). Specifically, this activation is mediated primarily through the recognition of LPS by Toll-like receptor 4 (TLR4) expressed on hepatic macrophages (Kupffer cells). This binding initiates a downstream signaling cascade that potently activates the NF-κB and JNK pathways, leading to the transcription and release of pro-inflammatory cytokines like TNF-α and IL-6. This chronic, low-grade inflammation is a key driver of hepatocyte injury, insulin resistance, and the progression from simple steatosis to NASH and fibrosis, potentially progressing to liver cancer over time.

Regulating the mechanisms of the gut-liver axis and its metabolites presents promising new avenues for treating MASLD and its associated liver cancer. Modifying the microbial community, strengthening gut barrier integrity, and utilizing metabolites such as SCFAs and bile acids may reduce liver inflammation, improve metabolic function, and contribute significantly to preventing and managing MASLD and its progression. These findings provide a crucial theoretical framework and practical direction for future clinical research and drug development.

### 6.3 The potential of gut microbiota intervention in improving metabolic disorders

Intervening with the gut microbiota holds considerable promise for the prevention and treatment of MASLD and its associated HCC. Recent studies have highlighted the close connection between changes in the gut microbiota and metabolic disorders, demonstrating their significant role in the onset and progression of MASLD. Interventions such as probiotics, dietary polysaccharides, and specific medications (e.g., Semaglutide) can effectively regulate the gut microbiota, thereby improving the metabolic status in MASLD. For instance, probiotics can enhance gut barrier function and modulate immune responses, potentially reducing liver inflammation and steatosis (118). Additionally, the consumption of dietary polysaccharides supports the growth of beneficial gut microbiota, leading to the production of SCFAs, which enhance insulin sensitivity and regulate hepatic lipid metabolism (119).

Semaglutide, a GLP-1 receptor agonist, has been investigated for its potential to improve the metabolic status of MASLD patients. It exerts its effects by regulating appetite, enhancing insulin secretion, and inhibiting hepatic glucose production, while also modulating the gut microbiota composition, promoting a more favorable metabolic profile (120). Studies have shown that Semaglutide treatment leads to a significant increase in gut microbiota diversity, with a notable rise in beneficial bacterial populations, further highlighting its potential for metabolic regulation. Modulating the gut microbiota not only helps improve metabolic disorders in MASLD but also prevents its progression to liver cancer. The progression of MASLD is closely associated with gut dysbiosis, inflammation, and alterations in the hepatic microenvironment. Through metabolites such as SCFAs and bile acids, the gut microbiota influences hepatic lipid metabolism and inflammation, playing a crucial role in maintaining liver health (121). Therefore, interventions targeting the gut microbiota, including probiotics, dietary polysaccharides, and pharmacological treatments, may provide novel strategies for the prevention and treatment of MASLD-related liver cancer. Moreover, studies have demonstrated that the interaction between the gut microbiota and inflammatory pathways, such as the NLRP3 inflammasome, plays a crucial role in MASLD and its progression. Modulating the gut microbiota may help reduce intestinal permeability and suppress inflammation, thereby alleviating liver damage and lowering the risk of liver cancer (122). Future research should focus on how gut microbiota interventions can enhance the metabolic status of MASLD patients and reduce the risk of liver cancer. Successfully implementing these interventions could offer valuable insights for the clinical management of MASLD and play a pivotal role in preventing and treating liver cancer.

To clarify the complex interactions between dysregulation of glucose and lipid metabolism, inflammatory pathways, and gut microbiota in the progression of MASLD-HCC, Table 1 offers a detailed summary of the key mechanisms and their roles in disease pathogenesis

**TABLE 1:** Key mechanisms of glucose and lipid metabolism dysregulation in non-alcoholic fatty liver disease-hepatocellular carcinoma (NAFLD-HCC).

Mechanism/process	Description	Role in NAFLD-HCC progression	References
Insulin resistance and abnormal glucose metabolism	Involves enhanced gluconeogenesis, impaired glucose utilization, and disrupted insulin signaling (e.g., via AMPK pathway). Leads to hyperglycemia and vicious cycle with lipid accumulation.	Promotes hepatic steatosis, inflammation, fibrosis, and carcinogenic microenvironment; key in NAFL to NASH transition without cirrhosis in ∼40%–50% cases.	(6, 7, 19, 20, 35–39)
Lipid accumulation and imbalanced fatty acid synthesis/oxidation	Upregulation of enzymes like FASN and ACC; impaired β-oxidation (e.g., suppressed CPT2). Involves metabolic reprogramming and oxidative stress.	Drives steatosis, lipotoxicity, cell apoptosis, and tumor cell survival in hypoxic environments; linked to genetic mutations (e.g., E2F1/E2F2 activation).	(22, 23, 27–31, 48–51)
Key signaling pathways (e.g., mTORC1, AMPK/ACC, FXR, NF-κB/JNK)	mTORC1 promotes lipid synthesis and proliferation; AMPK inhibits ACC to reduce fatty acid synthesis; FXR regulates bile acids and gut-liver axis; NF-κB/JNK drives inflammation and apoptosis.	Facilitates metabolic reprogramming, chronic inflammation, fibrosis, and HCC development; crosstalk exacerbates oxidative stress and gut dysbiosis effects.	(64–68, 70–72, 74–76, 81–86)
miRNA regulation (e.g., miR-22-3p) and post-translational modifications (e.g., O-GlcNAc)	miR-22-3p downregulation enhances glycolysis (Warburg effect); O-GlcNAc senses nutrients, regulates FASN and insulin signaling.	Exacerbates glucose intolerance, steatosis, and tumor growth; promotes insulin resistance and mitochondrial dysfunction in NAFLD progression.	(45–47, 98–106)
Gut microbiota dysbiosis and metabolites (e.g., SCFAs, bile acids)	Imbalance (e.g., increased Desulfovibrio) leads to barrier dysfunction, LPS translocation; metabolites like SCFAs influence insulin sensitivity and inflammation via gut-liver axis.	Induces hepatic inflammation, lipid deposition, and immune dysregulation; accelerates NAFLD to HCC via NLRP3 inflammasome and bile acid dysregulation.	(107–110, 112–117, 121, 122)

## 7 Clinical interventions and future prospects for glucose and lipid metabolism in masld-related hepatocellular carcinoma

### 7.1 Current drugs and progress in clinical trials

Metabolic dysfunction associated steatotic liver disease is the most prevalent chronic liver disease worldwide and is strongly linked to metabolic syndrome, making it a significant public health concern. While no specific drugs have been approved for MASLD treatment, advancements have been made in developing therapies that target its underlying pathological mechanisms. PPARα agonists, which regulate lipid and glucose metabolism, have shown promising results in clinical trials by significantly reducing liver fat content and improving liver function (123). GLP-1 receptor agonists, such as Semaglutide, effectively reduce liver fat accumulation and metabolic disturbances by enhancing insulin sensitivity and promoting weight loss (124). FGF21 analogs regulate energy and lipid metabolism, helping to reduce liver fat and improve insulin resistance. Clinical trials have shown good tolerability and safety, though the long-term efficacy is still being evaluated (125). In addition, inhibitors targeting the NLRP3 inflammasome help reduce liver inflammation and fibrosis, offering a promising new approach for MASLD treatment.

Recent updates bolster these agents’ profiles: Resmetirom (THR-β agonist) achieved FDA approval in 2024, with MAESTRO-NASH Phase 3 data confirming 26% NASH resolution and fibrosis reduction, though limitations include pruritus (15% incidence) and understudied HCC endpoints (126). Semaglutide’s ESSENCE phase 3 trial (72-week interim, AASLD 2024) showed 62.9% MASH resolution without fibrosis worsening (vs. 34.1% placebo; EDP 28.9%, *P* < 0.0001) and 37.0% fibrosis improvement without MASH progression (vs. 22.5%; EDP 14.5%, *P* < 0.0001), via GLP-1 metabolic/inflammatory effects and 10.5% weight loss, with 30%–40% liver enzyme/fibrosis marker reductions; GI AEs increased (nausea 36%, diarrhea 27%), and modest fibrosis gains highlight combo therapy potential (127). Lanifibranor’s NATIVE phase 2b trial (24-week data, 2024) yielded broad CMH improvements in MASH patients via pan-PPAR agonism, including 26% TG reduction (AMD −0.5 mmol/L), 0.5% HbA1c lowering, hs-CRP drops (−2.2 mg/L), and DBP decreases (−3.9 mmHg), with adiponectin surges (> 4-fold in 80%) correlating to histological MASH resolution (49%) and fibrosis regression (34%), independent of average 2.5 kg weight gain (128).

Given the limited efficacy of single-target drugs, multi-target combination therapies have emerged as a key area of research. For example, combining GLP-1 receptor agonists with SGLT2 inhibitors yields a synergistic effect, enhancing metabolic indicators and promoting liver health (129). This combined approach aims to improve treatment outcomes by addressing multiple pathological mechanisms, including metabolic regulation and inflammation. As our understanding of the underlying mechanisms of MASLD deepens, drug development targeting multiple pathways will be crucial for future therapies, providing more comprehensive treatment options for patients.

Although preclinical studies have shown promising results, translating these findings into clinical benefits remains a challenge. For instance, the SCD1 inhibitor Aramchol is currently being assessed in Phase III clinical trials for NASH and liver fibrosis, including the ARMOR study (130). As of mid-2025, however, no Phase III clinical trial results have been published for MASLD-related HCC patient subgroups, underscoring the critical need for focused clinical research in this specific population (131, 132). In the future, the development of novel drugs is expected to advance into clinical trials, offering new hope for the treatment of MASLD and its associated complications, such as HCC.

### 7.2 Emerging therapeutic targets and technologies

Gene editing and miRNA interventions have emerged as promising approaches for treating MASLD and HCC. Technologies such as CRISPR-Cas9 enable precise modification of genes linked to MASLD and HCC, facilitating the identification of pathogenic genes and the development of personalized treatments. Targeting key genes, such as AKT1, has demonstrated potential in improving lipid metabolism and inhibiting tumor growth in MASLD-related liver cancer (133). Additionally, miRNAs are crucial in regulating lipid metabolism, inflammation, and fibrosis. Targeting specific miRNAs may provide new therapeutic avenues for treating MASLD and HCC (134). O-GlcNAc modification, which impacts metabolic pathways, is also being investigated for its role in liver metabolic disorders and its potential contribution to the development of MASLD (135). Furthermore, mTORC1 inhibitors are being investigated for their potential to improve lipid metabolism and reduce liver inflammation in MASLD. Inhibiting mTORC1 has shown promise in preventing the progression of MASLD to HCC by mitigating lipid accumulation and inflammation (136). However, challenges such as potential side effects and the complexity of the mTORC1 pathway persist. Further research is required to develop more targeted treatments that modulate mTORC1, ensuring a balance between efficacy and safety in clinical settings.

In conclusion, the integration of gene editing, miRNA intervention, O-GlcNAc modification regulation, and mTORC1 inhibition presents promising new strategies for treating MASLD and HCC. Ongoing research will refine these approaches, opening up new possibilities for clinical management.

### 7.3 Future research directions

Understanding the roles of these networks in the progression of MASLD and HCC requires a comprehensive analysis using advanced technologies such as metabolomics, transcriptomics, and single-cell RNA sequencing. These approaches can provide insights into how glucose-lipid metabolism influences hepatocyte biology and tumorigenesis (137). Personalized treatment strategies will be essential moving forward, as MASLD and liver cancer patients display considerable heterogeneity in metabolic profiles, genetics, and lifestyle. Integrating genomics and epigenetics can help identify genetic susceptibilities linked to MASLD, allowing for personalized prevention and treatment plans tailored to high-risk individuals (138). Additionally, designing interventions tailored to metabolic states—such as diet, exercise, and pharmacotherapy—will open up new avenues for managing MASLD. Integrating multi-omics is crucial for uncovering the metabolic heterogeneity of MASLD and its progression to liver cancer. Combining data from genomics, transcriptomics, metabolomics, and proteomics can help identify novel biomarkers for precision diagnosis and treatment. Additionally, exploring interactions between hepatocytes and their microenvironment, including immune and stellate cells, will shed light on their roles in the progression of MASLD and HCC (139).

To advance the field, future research should address key questions and prioritize targeted proposals. These include: (1) Biomarker-Guided Therapy: Can baseline serum FGF21 levels or circulating miR-22-3p expression profiles predict patient responses to metabolic therapies, enabling personalized treatment? This could involve clinical trials evaluating FGF21 as a biomarker for MASLD severity, guiding analog dosing to prevent HCC in high-risk patients, and using miR-22-3p for stratifying those with metabolic reprogramming to enable targeted miRNA therapies in glycolytic-dominant HCC. (2) Non-invasive Risk Stratification: How can multi-omics data—integrating genomics, metabolomics, and gut microbiome signatures—develop robust algorithms to stratify MASLD patients by HCC risk, optimizing surveillance? Specific queries, such as whether FGF21/miR-22-3p panels improve early HCC detection in MASLD cohorts, provide a roadmap for validation. (3) Causal Mechanisms of the Gut-Liver Axis: What are the roles of microbial-derived metabolites (e.g., bile acids or SCFAs) in hepatocyte transformation, and can interventions like engineered probiotics or postbiotics modify these pathways to prevent HCC? Finally, as the interactions between gut microbiota and host metabolism receive increasing attention, Table 2 summarizes emerging therapeutic targets and interventions for MASLD-HCC, emphasizing their potential to modulate glucose and lipid metabolism and reduce inflammation.

**TABLE 2:** Emerging therapeutic targets and interventions for glucose and lipid metabolism in non-alcoholic fatty liver disease-hepatocellular carcinoma (NAFLD-HCC).

Target/intervention	Description	Potential benefits and evidence	References
Target/intervention description potential benefits and evidence references PPARα agonists (e.g., fenofibrate)	Nuclear receptor promoting fatty acid β-oxidation via CPT1A/ACOX1; inhibits inflammation and fibrosis.	Reduces steatosis, improves lipid metabolism, and prevents NASH progression; clinical trials show lipid-lowering and anti-fibrotic effects.	(52–62, 123)
FGF21 analogs (e.g., Pegbelfermin)	Hormone enhancing insulin sensitivity and reducing hepatic fat; regulates lipid metabolism and acts as biomarker.	Alleviates NAFLD severity, improves metabolic health; Phase II trials demonstrate safety, reduced steatosis, and potential HCC prevention.	(90–97, 125)
GLP-1 receptor agonists (e.g., Semaglutide)	Promotes insulin secretion, weight loss; modulates gut microbiota and SCFAs production.	Improves insulin resistance, reduces liver fat/inflammation; reshapes microbiota for better gut-liver axis function; synergistic in combination therapies.	(120, 124, 129)
mTORC1 inhibitors (e.g., rapamycin) and AMPK activators (e.g., 5-ALA)	mTORC1 inhibition reduces lipid synthesis and proliferation; AMPK activation inhibits ACC, promotes oxidation.	Restricts HCC growth, ameliorates steatosis/inflammation; preclinical models show reduced tumor progression in NAFLD contexts.	(69, 73, 74, 136)
Gut microbiota modulation (e.g., probiotics, prebiotics, FMT)	Targets dysbiosis via polysaccharides or drugs; enhances barrier integrity and metabolite production (SCFAs, bile acids).	Alleviates inflammation, improves metabolic disorders; prevents NAFLD-HCC progression; emerging for adjunct therapy with FXR agonists.	(111, 118, 119, 121, 122, 126)
Gene/miRNA editing and multi-omics approaches	CRISPR-Cas9 for genes like AKT1; miRNA interventions; integrates metabolomics/transcriptomics for biomarkers.	Enables personalized targeting of metabolic heterogeneity; identifies HCC risk in NAFLD; future for precision diagnostics/therapies.	(133–135, 137–139)

## 8 Conclusion

**FIGURE 1:**
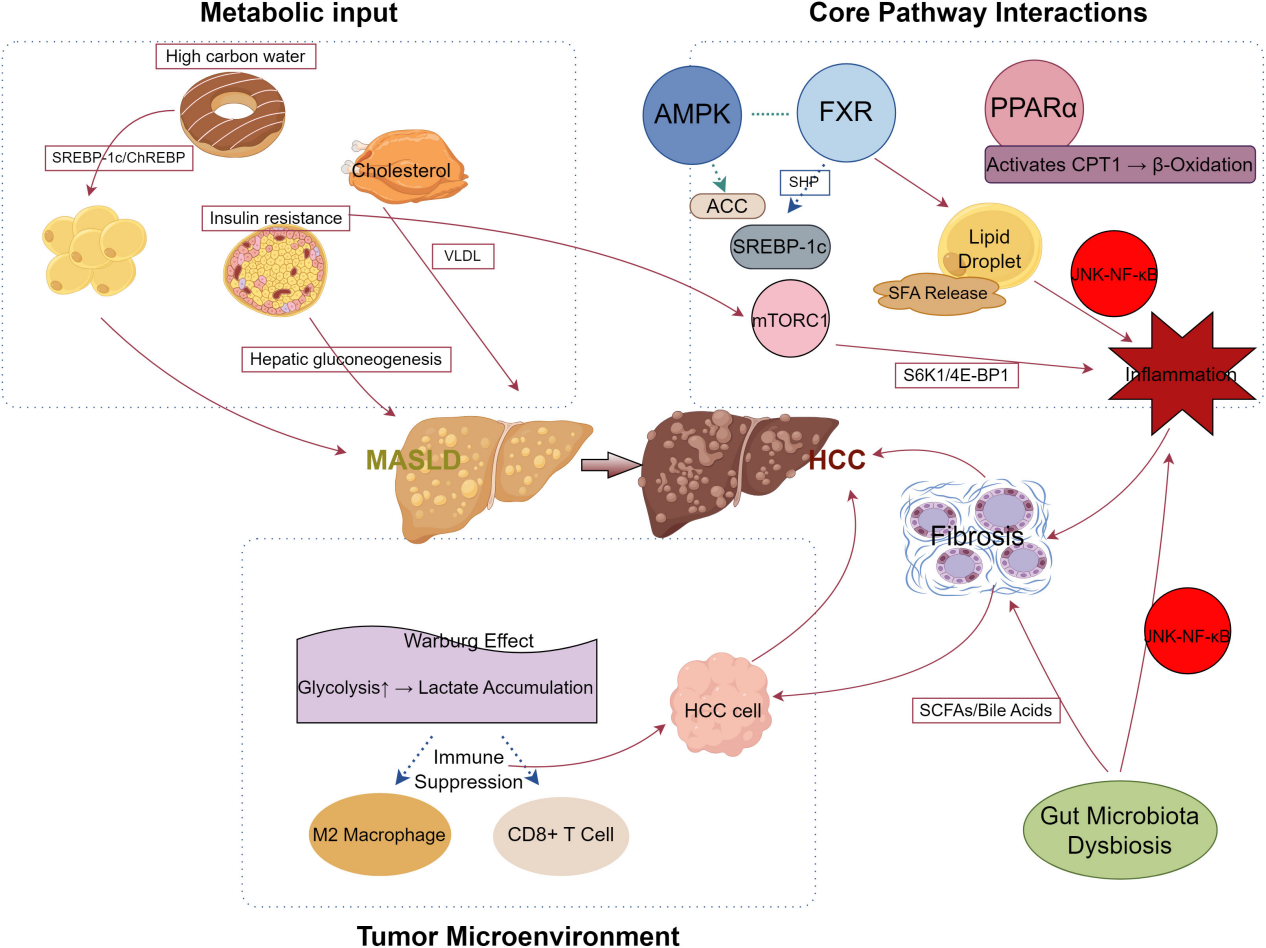
Mechanistic pathway of metabolic dysfunction-associated steatotic liver disease (MASLD) progression to hepatocellular carcinoma (HCC). This diagram illustrates the metabolic and molecular mechanisms driving the progression from MASLD to HCC. The liver is depicted centrally, with surrounding quadrants representing key processes: (1) Metabolic Input (left) highlights dietary and physiological inputs; (2) Core Pathway Interactions (upper right) shows signaling pathways; and (3) Tumor Microenvironment (lower right) depicts the tumor-supportive environment. Key elements include: high carbohydrate intake and cholesterol promoting SREBP-1c/ChREBP and VLDL secretion, respectively, exacerbated by insulin resistance and hepatic gluconeogenesis leading to MASLD; AMP-activatedproteinkinase (AMPK) as an upstream regulator phosphorylating and inhibiting ACC; farnesoid X receptor (FXR) and PPARα regulating lipid metabolism via SHP and CPT1-mediated β-oxidation, while mTORC1 activates S6K1/4E-BP1 and SREBP-1c, enhancing lipid droplet formation and inflammation through JNK-NF-κB; and the Warburg effect driving glycolysis and lactate accumulation, fostering HCC cell growth, fibrosis, and an immunosuppressive microenvironment with M2 macrophages and suppressed CD8+ T cells. Gut microbiota dysbiosis alters the pool of short-chainfattyacids (SCFAs) and bile acids, which activates JNK-NF-κB to amplify inflammation and/or fibrosis. Red arrow lines indicate promotion or enhancement (e.g., activation of pathways or processes), while blue arrow lines denote inhibition (e.g., suppression of immune responses or pathways).

In conclusion, studies on MASLD-related liver cancer is advancing rapidly, revealing the complex interactions between glucose-lipid metabolism disturbances, key regulatory factors, and the gut microbiota. These insights are crucial not only for deepening our understanding of the disease’s pathophysiology but also for developing novel therapeutic approaches. Figure 1 presents a succinct visual overview of these mechanisms, showing how metabolic disruptions initiate MASLD, how core pathway interactions intensify inflammation and lipid accumulation, and how the tumor microenvironment contributes to HCC development. The pivotal role of metabolic dysregulation in driving liver fat accumulation, hepatocellular carcinoma formation, and disease progression emphasizes the importance of comprehensive strategies for treatment and prevention. Targeting key molecules such as PPARα, FGF21, miRNAs, and O-GlcNAc modification holds promise for therapeutic interventions, positioning metabolic regulation as a central strategy for innovative treatments.

Furthermore, the impact of the gut microbiota on liver metabolism and disease progression represents a promising area of research, indicating that modulating the microbiome could serve as a valuable complement to conventional therapies. Gaining a deeper understanding of the bidirectional interaction between the liver and the gut microbiome will be crucial for developing more effective intervention strategies. Additionally, the emergence of precision medicine, fueled by the integration of genomic, metabolic, and microbiome data, will facilitate the tailoring of treatments to individual patient profiles, ultimately improving therapeutic outcomes.

Despite significant advancements, challenges persist in translating these findings into clinical practice, especially regarding early diagnosis and personalized treatment. Current limitations include therapeutic safety concerns, such as pruritus and adverse lipid changes with FXR agonists like obeticholic acid, and uncertainties in long-term efficacy for emerging drugs like FGF21 analogs, which show short-term fibrosis reductions but require extended trials to confirm HCC prevention. Gaps also exist in addressing patient heterogeneity and comorbidities, which may reduce treatment responses and complicate real-world application. Future research should prioritize exploring the complexities of glucose-lipid metabolic networks, their interactions with the gut microbiota, and their roles in the progression of MASLD and liver cancer. By leveraging multi-omics technologies and deepening our understanding of underlying mechanisms, it will be possible to identify novel biomarkers for early detection, predict disease progression, and develop more effective, tailored treatment strategies. Ultimately, integrating these insights offers great potential for improving patient outcomes and quality of life, providing a comprehensive approach to managing MASLD-related liver cancer.

## References

[1] Rinella M Lazarus J Ratziu V Francque S Sanyal A Kanwal F A multisociety Delphi consensus statement on new fatty liver disease nomenclature. *Hepatology*. (2023) 78:1966–86. 10.1097/HEP.0000000000000520 37363821 PMC10653297

[2] European Association for the Study of the Liver (EASL), European Association for the Study of Diabetes (EASD), European Association for the Study of Obesity (EASO). EASL-EASD-EASO Clinical Practice Guidelines on the management of metabolic dysfunction-associated steatotic liver disease (MASLD). *J Hepatol*. (2024) 81:492–542. 10.1016/j.jhep.2024.04.031 38851997

[3] Cespiati A Cinque F Meroni M Lombardi R Dongiovanni P Fracanzani A. An overview of hepatocellular carcinoma surveillance focusing on non-cirrhotic NAFLD patients: a challenge for physicians. *Biomedicines*. (2023) 11:586. 10.3390/biomedicines11020586 36831120 PMC9953185

[4] Tang J Zheng N Yan Y Zhang N Ren X. 1990-2021 global, regional, and national analysis of the burden and trends of non-alcoholic fatty liver disease. *Front Med*. (2025) 12:1609816. 10.3389/fmed.2025.1609816 40547911 PMC12179171

[5] Samji N Verma R Satapathy S. Magnitude of nonalcoholic fatty liver disease: western perspective. *J Clin Exp Hepatol*. (2019) 9:497–505. 10.1016/j.jceh.2019.05.001 31516266 PMC6728535

[6] Samy A Kandeil M Sabry D Abdel-Ghany A Mahmoud M. From NAFLD to NASH: understanding the spectrum of non-alcoholic liver diseases and their consequences. *Heliyon*. (2024) 10:e30387. 10.1016/j.heliyon.2024.e30387 38737288 PMC11088336

[7] Zhang Q Guo J Zhou L Dong H. [Research progress of non-alcoholic fatty liver disease in postmenopausal women]. *Zhonghua Gan Zang Bing Za Zhi.* (2020) 28:629–32. 10.3760/cma.j.cn501113-20200525-00270 32791802 PMC12769300

[8] Tran D Double K Johnston I Westbrook R Harris I. Consumption of a diet high in fat and sugar is associated with worse spatial navigation ability in a virtual environment. *Int J Obes*. (2025) 49:1354–62. 10.1038/s41366-025-01776-8 40247089 PMC12283396

[9] Stanhope K. Sugar consumption, metabolic disease and obesity: the state of the controversy. *Crit Rev Clin Lab Sci*. (2016) 53:52–67. 10.3109/10408363.2015.1084990 26376619 PMC4822166

[10] Chen X Liu M Tang J Wang N Feng Y Ma H. Research progress on the therapeutic effect of polysaccharides on non-alcoholic fatty liver disease through the regulation of the gut-liver axis. *Int J Mol Sci*. (2022) 23:11710. 10.3390/ijms231911710 36233011 PMC9570256

[11] Tilg H Adolph T Dudek M Knolle P. Non-alcoholic fatty liver disease: the interplay between metabolism, microbes and immunity. *Nat Metab*. (2021) 3:1596–607. 10.1038/s42255-021-00501-9 34931080

[12] Shah P Patil R Harrison SA. NAFLD-related hepatocellular carcinoma: the growing challenge. *Hepatology*. (2023) 77:323–38. 10.1002/hep.32542 35478412 PMC9970023

[13] Hagström H Kechagias S Ekstedt M. Risk for hepatic and extra-hepatic outcomes in nonalcoholic fatty liver disease. *J Intern Med*. (2022) 292:177–89. 10.1111/joim.13343 34118091

[14] Agyapong G Dashti F Banini B. Nonalcoholic liver disease: epidemiology, risk factors, natural history, and management strategies. *Ann N Y Acad Sci*. (2023) 1526:16–29. 10.1111/nyas.15012 37400359 PMC10524684

[15] Park M Rahman M Rahman M Kim J Choi M Kim J Potential therapeutic implication of herbal medicine in mitochondria-mediated oxidative stress-related liver diseases. *Antioxidants*. (2022) 11:2041. 10.3390/antiox11102041 36290765 PMC9598588

[16] Kim H Park C Kim T. Targeting liver X receptors for the treatment of non-alcoholic fatty liver disease. *Cells*. (2023) 12:1292. 10.3390/cells12091292 37174692 PMC10177243

[17] Thomas J Kendall B El-Serag H Thrift A Macdonald G. Hepatocellular and extrahepatic cancer risk in people with non-alcoholic fatty liver disease. *Lancet Gastroenterol Hepatol*. (2024) 9:159–69. 10.1016/S2468-1253(23)00275-3 38215780

[18] Zambrano-Vasquez O Cortes-Camacho F Castaneda-Sanchez J Arechaga-Ocampo E Valle-Velazquez E Cabrera-Angeles J Update in non-alcoholic fatty liver disease management: role of sodium-glucose cotransporter 2 inhibitors. *Life Sci.* (2025) 372:123638. 10.1016/j.lfs.2025.123638 40246191

[19] Loomba R Friedman S Shulman G. Mechanisms and disease consequences of nonalcoholic fatty liver disease. *Cell*. (2021) 184:2537–64. 10.1016/j.cell.2021.04.015 33989548 PMC12168897

[20] Palma R Pronio A Romeo M Scognamiglio F Ventriglia L Ormando V The role of insulin resistance in fueling NAFLD pathogenesis: from molecular mechanisms to clinical implications. *J Clin Med*. (2022) 11:3649. 10.3390/jcm11133649 35806934 PMC9267803

[21] Saeed A Dullaart R Schreuder T Blokzijl H Faber K. Disturbed vitamin A metabolism in non-alcoholic fatty liver disease (NAFLD). *Nutrients*. (2017) 10:29. 10.3390/nu10010029 29286303 PMC5793257

[22] Huh J Saltiel A. Roles of IκB kinases and TANK-binding kinase 1 in hepatic lipid metabolism and nonalcoholic fatty liver disease. *Exp Mol Med*. (2021) 53:1697–705. 10.1038/s12276-021-00712-w 34848839 PMC8639992

[23] Huang J Sigon G Mullish B Wang D Sharma R Manousou P Applying lipidomics to non-alcoholic fatty liver disease: a clinical perspective. *Nutrients*. (2023) 15:1992. 10.3390/nu15081992 37111211 PMC10143024

[24] Baffy G Brunt E Caldwell S. Hepatocellular carcinoma in non-alcoholic fatty liver disease: an emerging menace. *J Hepatol*. (2012) 56:1384–91. 10.1016/j.jhep.2011.10.027 22326465

[25] Piek A Smit L Suthahar N Bakker S de Boer R Silljé H. The emerging plasma biomarker Dickkopf-3 (DKK3) and its association with renal and cardiovascular disease in the general population. *Sci Rep*. (2021) 11:8642. 10.1038/s41598-021-88107-9 33883651 PMC8060267

[26] Xie L Wang P Zhang P Zhang X Zhao G Wang A DKK3 expression in hepatocytes defines susceptibility to liver steatosis and obesity. *J Hepatol*. (2016) 65:113–24. 10.1016/j.jhep.2016.03.008 27016281

[27] Sunami Y. NASH, fibrosis and hepatocellular carcinoma: lipid synthesis and glutamine/Acetate signaling. *Int J Mol Sci*. (2020) 21:6799. 10.3390/ijms21186799 32947972 PMC7555727

[28] González-Romero F Mestre D Aurrekoetxea I O’Rourke C Andersen J Woodhoo A E2F1 and E2F2-mediated repression of CPT2 establishes a lipid-rich tumor-promoting environment. *Cancer Res*. (2021) 81:2874–87. 10.1158/0008-5472.CAN-20-2052 33771899

[29] Qi F Li J Qi Z Zhang J Zhou B Yang B Comprehensive metabolic profiling and genome-wide analysis reveal therapeutic modalities for hepatocellular carcinoma. *Research*. (2023) 6:0036. 10.34133/research.0036 37040510 PMC10076022

[30] Li L Guo Z Zhao Y Liang C Zheng W Tian W The impact of oxidative stress on abnormal lipid metabolism-mediated disease development. *Arch Biochem Biophys*. (2025) 766:110348. 10.1016/j.abb.2025.110348 39961502

[31] González P Lozano P Ros G Solano F. Hyperglycemia and oxidative stress: an integral, updated and critical overview of their metabolic interconnections. *Int J Mol Sci*. (2023) 24:9352. 10.3390/ijms24119352 37298303 PMC10253853

[32] Guo Z Fan X Yao J Tomlinson S Yuan G He S. The role of complement in nonalcoholic fatty liver disease. *Front Immunol*. (2022) 13:1017467. 10.3389/fimmu.2022.1017467 36248852 PMC9562907

[33] Shi J Fan J Su Q Yang Z. Cytokines and abnormal glucose and lipid metabolism. *Front Endocrinol*. (2019) 10:703. 10.3389/fendo.2019.00703 31736870 PMC6833922

[34] Kim H Yoo Y. The role of STAMP2 in pathogenesis of chronic diseases focusing on nonalcoholic fatty liver disease: a review. *Biomedicines*. (2022) 10:2082. 10.3390/biomedicines10092082 36140186 PMC9495648

[35] Bessone F Razori M Roma M. Molecular pathways of nonalcoholic fatty liver disease development and progression. *Cell Mol Life Sci*. (2019) 76:99–128. 10.1007/s00018-018-2947-0 30343320 PMC11105781

[36] Chen C Hsu L Chen K Chiu K Chen C Huang K. Emerging roles of calcium signaling in the development of non-alcoholic fatty liver disease. *Int J Mol Sci*. (2021) 23:256. 10.3390/ijms23010256 35008682 PMC8745268

[37] Li Y Xin L Zhao Y Li S Li Y. Role of vascular endothelial growth factor B in nonalcoholic fatty liver disease and its potential value. *World J Hepatol*. (2023) 15:786–96. 10.4254/wjh.v15.i6.786 37397934 PMC10308292

[38] Zheng Z Li Y Fan S An J Luo X Liang M WW domain-binding protein 2 overexpression prevents diet-induced liver steatosis and insulin resistance through AMPKβ1. *Cell Death Dis*. (2021) 12:228. 10.1038/s41419-021-03536-8 33658485 PMC7930037

[39] Fang C Pan J Qu N Lei Y Han J Zhang J The AMPK pathway in fatty liver disease. *Front Physiol*. (2022) 13:970292. 10.3389/fphys.2022.970292 36203933 PMC9531345

[40] Li Y Li W Zhu X Xu N Meng Q Jiang W VEGFB ameliorates insulin resistance in NAFLD via the PI3K/AKT signal pathway. *J Transl Med*. (2024) 22:976. 10.1186/s12967-024-05621-w 39468621 PMC11520811

[41] Vily-Petit J Soty-Roca M Silva M Raffin M Gautier-Stein A Rajas F Intestinal gluconeogenesis prevents obesity-linked liver steatosis and non-alcoholic fatty liver disease. *Gut*. (2020) 69:2193–202. 10.1136/gutjnl-2019-319745 32205419

[42] Lu Q Tian X Wu H Huang J Li M Mei Z Metabolic changes of hepatocytes in NAFLD. *Front Physiol*. (2021) 12:710420. 10.3389/fphys.2021.710420 34526911 PMC8437340

[43] Li S Hao L Hu X. Natural products target glycolysis in liver disease. *Front Pharmacol*. (2023) 14:1242955. 10.3389/fphar.2023.1242955 37663261 PMC10469892

[44] Pan G Cavalli M Wadelius C. Polymorphisms rs55710213 and rs56334587 regulate SCD1 expression by modulating HNF4A binding. *Biochim Biophys Acta Gene Regul Mech*. (2021) 1864:194724. 10.1016/j.bbagrm.2021.194724 34171462

[45] Gjorgjieva M Sobolewski C Ay A Abegg D Correia de Sousa M Portius D Genetic ablation of MiR-22 fosters diet-induced obesity and NAFLD development. *J Pers Med*. (2020) 10:170. 10.3390/jpm10040170 33066497 PMC7711493

[46] Yang F Hu Y Liu H Wan Y. MiR-22-silenced cyclin A expression in colon and liver cancer cells is regulated by bile acid receptor. *J Biol Chem*. (2015) 290:6507–15. 10.1074/jbc.M114.620369 25596928 PMC4358284

[47] Wang D Sang Y Sun T Kong P Zhang L Dai Y Emerging roles and mechanisms of microRNA-222-3p in human cancer (Review). *Int J Oncol*. (2021) 58:20. 10.3892/ijo.2021.5200 33760107 PMC7979259

[48] Fernando D Forbes J Angus P Herath C. Development and progression of non-alcoholic fatty liver disease: the role of advanced glycation end products. *Int J Mol Sci*. (2019) 20:5037. 10.3390/ijms20205037 31614491 PMC6834322

[49] O’Farrell M Duke G Crowley R Buckley D Martins E Bhattacharya D FASN inhibition targets multiple drivers of NASH by reducing steatosis, inflammation and fibrosis in preclinical models. *Sci Rep*. (2022) 12:15661. 10.1038/s41598-022-19459-z 36123383 PMC9485253

[50] Dorn C Riener M Kirovski G Saugspier M Steib K Weiss T Expression of fatty acid synthase in nonalcoholic fatty liver disease. *Int J Clin Exp Pathol*. (2010) 3:505–14. 20606731 PMC2897101

[51] Xu H Wan S An Y Wu Q Xing Y Deng C Targeting cell death in NAFLD: mechanisms and targeted therapies. *Cell Death Discov*. (2024) 10:399. 10.1038/s41420-024-02168-z 39244571 PMC11380694

[52] Iturbe-Rey S Maccali C Arrese M Aspichueta P Oliveira C Castro R Lipotoxicity-driven metabolic dysfunction-associated steatotic liver disease (MASLD). *Atherosclerosis*. (2025) 400:119053. 10.1016/j.atherosclerosis.2024.119053 39581063

[53] Chen H Qi X Guan K Wang R Li Q Ma Y. Tandem mass tag-based quantitative proteomics analysis reveals the effects of the α-lactalbumin peptides GINY and DQW on lipid deposition and oxidative stress in HepG2 cells. *J Dairy Sci*. (2023) 106:2271–88. 10.3168/jds.2022-22511 36797178

[54] Yan T Luo Y Yan N Hamada K Zhao N Xia Y Intestinal peroxisome proliferator-activated receptor α-fatty acid-binding protein 1 axis modulates nonalcoholic steatohepatitis. *Hepatology*. (2023) 77:239–55. 10.1002/hep.32538 35460276 PMC9970020

[55] Zhong J He X Gao X Liu Q Zhao Y Hong Y Hyodeoxycholic acid ameliorates nonalcoholic fatty liver disease by inhibiting RAN-mediated PPARα nucleus-cytoplasm shuttling. *Nat Commun*. (2023) 14:5451. 10.1038/s41467-023-41061-8 37673856 PMC10482907

[56] Zhu X Liu Q Patterson A Sharma A Amin S Cohen S Accumulation of linoleic acid by altered peroxisome proliferator-activated receptor-α signaling is associated with age-dependent hepatocarcinogenesis in Ppara transgenic mice. *Metabolites*. (2023) 13:936. 10.3390/metabo13080936 37623879 PMC10456914

[57] Lin Y Wang Y Li PF. PPARα: an emerging target of metabolic syndrome, neurodegenerative and cardiovascular diseases. *Front Endocrinol*. (2022) 13:1074911. 10.3389/fendo.2022.1074911 36589809 PMC9800994

[58] Hu P Li K Peng X Kan Y Li H Zhu Y Nuclear receptor PPARα as a therapeutic target in diseases associated with lipid metabolism disorders. *Nutrients*. (2023) 15:4772. 10.3390/nu15224772 38004166 PMC10674366

[59] Theys C Vanderhaeghen T Van Dijck E Peleman C Scheepers A Ibrahim J Loss of PPARα function promotes epigenetic dysregulation of lipid homeostasis driving ferroptosis and pyroptosis lipotoxicity in metabolic dysfunction associated Steatotic liver disease (MASLD). *Front Mol Med*. (2023) 3:1283170. 10.3389/fmmed.2023.1283170 39086681 PMC11285560

[60] Clavreul L Bernard L Cotte A Hennuyer N Bourouh C Devos C The ubiquitin-like modifier FAT10 is induced in MASLD and impairs the lipid-regulatory activity of PPARα. *Metabolism*. (2024) 151:155720. 10.1016/j.metabol.2023.155720 37926201

[61] Zhu X Hou Q Zhang L Wang D Tian Z Liu Y Isorhynchophylline improves lipid metabolism disorder by mediating a circadian rhythm gene Bmal1 in spontaneously hypertensive rat. *Phytother Res*. (2023) 37:5991–6005. 10.1002/ptr.8015 37752617

[62] Barb D Kalavalapalli S Godinez Leiva E Bril F Huot-Marchand P Dzen L Pan-PPAR agonist lanifibranor improves insulin resistance and hepatic steatosis in patients with T2D and MASLD. *J Hepatol*. (2025) 82:979–91. 10.1016/j.jhep.2024.12.045 39824443

[63] Cooreman M Vonghia L Francque SM. MASLD/MASH and type 2 diabetes: two sides of the same coin? From single PPAR to pan-PPAR agonists. *Diabetes Res Clin Pract*. (2024) 212:111688. 10.1016/j.diabres.2024.111688 38697298

[64] Shiragannavar V Sannappa Gowda N Puttahanumantharayappa L Karunakara S Bhat S Prasad S The ameliorating effect of withaferin A on high-fat diet-induced non-alcoholic fatty liver disease by acting as an LXR/FXR dual receptor activator. *Front Pharmacol*. (2023) 14:1135952. 10.3389/fphar.2023.1135952 36909161 PMC9995434

[65] Zhang W Feng L Li P Wang A Dai C Qi Y Effects of Mao tea from Nankun Mountain on nonalcoholic fatty liver disease in mice. *Food Funct*. (2024) 15:9863–79. 10.1039/d4fo01689k 39246047

[66] Wang W Qin J Bai S Tian J Zhou Y Qin X Integrative transcriptomics and lipidomics unravels the amelioration effects of Radix Bupleuri on non-alcoholic fatty liver disease. *J Ethnopharmacol*. (2025) 338:119005. 10.1016/j.jep.2024.119005 39490432

[67] Li C Ni S Zhao L Lin H Yang X Zhang Q Effects of PM2.5 and high-fat diet on glucose and lipid metabolisms and role of MT-COX3 methylation in male rats. *Environ Int*. (2024) 188:108780. 10.1016/j.envint.2024.108780 38821017

[68] Hefer M Petrovic A Roguljic L Kolaric T Kizivat T Wu C Green tea polyphenol (-)-Epicatechin pretreatment mitigates hepatic steatosis in an in vitro MASLD model. *Curr Issues Mol Biol*. (2024) 46:8981–94. 10.3390/cimb46080531 39194748 PMC11352320

[69] Tian T Li X Zhang J. mTOR signaling in cancer and mTOR inhibitors in solid tumor targeting therapy. *Int J Mol Sci*. (2019) 20:755. 10.3390/ijms20030755 30754640 PMC6387042

[70] Hsu C Peng D Cai Z Lin HK. AMPK signaling and its targeting in cancer progression and treatment. *Semin Cancer Biol*. (2022) 85:52–68. 10.1016/j.semcancer.2021.04.006 33862221 PMC9768867

[71] An H Jang Y Choi J Hur J Kim S Kwon Y. New insights into AMPK, as a potential therapeutic target in metabolic dysfunction-associated steatotic liver disease and hepatic fibrosis. *Biomol Ther*. (2025) 33:18–38. 10.4062/biomolther.2024.188 39702310 PMC11704404

[72] Chen Y He X Chen X Li Y Ke Y. SeP is elevated in NAFLD and participates in NAFLD pathogenesis through AMPK/ACC pathway. *J Cell Physiol*. (2021) 236:3800–7. 10.1002/jcp.30121 33094480

[73] Yu H Zhang M Ma Y Lu J Pan J Pan P 5-ALA ameliorates hepatic steatosis through AMPK signaling pathway. *J Mol Endocrinol*. (2017) 59:121–8. 10.1530/JME-16-0260 28566408

[74] Lu T Sun L Wang Z Zhang Y He Z Xu C. Fatty acid synthase enhances colorectal cancer cell proliferation and metastasis via regulating AMPK/mTOR pathway. *Onco Targets Ther*. (2019) 12:3339–47. 10.2147/OTT.S199369 31118685 PMC6504633

[75] Li J Huang Q Long X Zhang J Huang X Aa J CD147 reprograms fatty acid metabolism in hepatocellular carcinoma cells through Akt/mTOR/SREBP1c and P38/PPARα pathways. *J Hepatol*. (2015) 63:1378–89. 10.1016/j.jhep.2015.07.039 26282231

[76] Stofan M Guo G. Bile acids and FXR: novel targets for liver diseases. *Front Med*. (2020) 7:544. 10.3389/fmed.2020.00544 33015098 PMC7516013

[77] Shah R Kowdley K. Obeticholic acid for the treatment of nonalcoholic steatohepatitis. *Expert Rev Gastroenterol Hepatol*. (2020) 14:311–21. 10.1080/17474124.2020.1748498 32241197

[78] Lambrecht J van Grunsven L Tacke F. Current and emerging pharmacotherapeutic interventions for the treatment of liver fibrosis. *Expert Opin Pharmacother*. (2020) 21:1637–50. 10.1080/14656566.2020.1774553 32543284

[79] Said I Ahad H Said A. Gut microbiome in non-alcoholic fatty liver disease associated hepatocellular carcinoma: current knowledge and potential for therapeutics. *World J Gastrointest Oncol*. (2022) 14:947–58. 10.4251/wjgo.v14.i5.947 35646285 PMC9124992

[80] Helmstädter M Schmidt J Kaiser A Weizel L Proschak E Merk D. Differential therapeutic effects of FXR activation, sEH inhibition, and dual FXR/sEH modulation in NASH in diet-induced obese mice. *ACS Pharmacol Transl Sci*. (2021) 4:966–79. 10.1021/acsptsci.1c00041 33860214 PMC8033765

[81] Czauderna C Castven D Mahn F Marquardt J. Context-dependent role of NF-κB signaling in primary liver cancer-from tumor development to therapeutic implications. *Cancers*. (2019) 11:1053. 10.3390/cancers11081053 31349670 PMC6721782

[82] Yu H Lin L Zhang Z Zhang H Hu H. Targeting NF-κB pathway for the therapy of diseases: mechanism and clinical study. *Signal Transduct Target Ther*. (2020) 5:209. 10.1038/s41392-020-00312-6 32958760 PMC7506548

[83] Li X Li S Li N. Research progress on natural products alleviating liver inflammation and fibrosis via NF-κB pathway. *Chem Biodivers*. (2025) 22:e202402248. 10.1002/cbdv.202402248 39576739

[84] Tao L Ren X Zhai W Chen Z. Progress and prospects of non-canonical NF-κB signaling pathway in the regulation of liver diseases. *Molecules*. (2022) 27:4275. 10.3390/molecules27134275 35807520 PMC9268066

[85] Ruan J Qi Z Shen L Jiang Y Xu Y Lan L Crosstalk between JNK and NF-κB signaling pathways via HSP27 phosphorylation in HepG2 cells. *Biochem Biophys Res Commun*. (2015) 456:122–8. 10.1016/j.bbrc.2014.11.045 25446109

[86] Mu W Cheng X Zhang X Liu Y Lv Q Liu G Hinokiflavone induces apoptosis via activating mitochondrial ROS/JNK/caspase pathway and inhibiting NF-κB activity in hepatocellular carcinoma. *J Cell Mol Med*. (2020) 24:8151–65. 10.1111/jcmm.15474 32519392 PMC7348176

[87] Khan H Ullah H Castilho P Gomila A D’Onofrio G Filosa R Targeting NF-κB signaling pathway in cancer by dietary polyphenols. *Crit Rev Food Sci Nutr*. (2020) 60:2790–800. 10.1080/10408398.2019.1661827 31512490

[88] Wang Y. Attenuation of berberine on lipopolysaccharide-induced inflammatory and apoptosis responses in β-cells via TLR4-independent JNK/NF-κB pathway. *Pharm Biol*. (2013): 10.3109/13880209.2013.840851 Online ahead of print. 24188583

[89] Zhao N Wang R Zhou L Zhu Y Gong J Zhuang S. MicroRNA-26b suppresses the NF-κB signaling and enhances the chemosensitivity of hepatocellular carcinoma cells by targeting TAK1 and TAB3. *Mol Cancer*. (2014) 13:35. 10.1186/1476-4598-13-35 24565101 PMC3938074

[90] Falamarzi K Malekpour M Tafti M Azarpira N Behboodi M Zarei M. The role of FGF21 and its analogs on liver associated diseases. *Front Med*. (2022) 9:967375. 10.3389/fmed.2022.967375 36457562 PMC9705724

[91] Negroiu C Tudoraşcu R Beznă M Ungureanu A Honţaru S Dănoiu S. The role of FGF21 in the interplay between obesity and non-alcoholic fatty liver disease: a narrative review. *Rom J Morphol Embryol*. (2024) 65:159–72. 10.47162/RJME.65.2.02 39020530 PMC11384831

[92] Zheng Q Martin R Shi X Pandit H Yu Y Liu X Lack of FGF21 promotes NASH-HCC transition via hepatocyte-TLR4-IL-17A signaling. *Theranostics*. (2020) 10:9923–36. 10.7150/thno.45988 32929325 PMC7481424

[93] Verzijl C Van De Peppel I Struik D Jonker J. Pegbelfermin (BMS-986036): an investigational PEGylated fibroblast growth factor 21 analogue for the treatment of nonalcoholic steatohepatitis. *Expert Opin Investig Drugs*. (2020) 29:125–33. 10.1080/13543784.2020.1708898 31899984

[94] Keinicke H Sun G Mentzel C Fredholm M John L Andersen B FGF21 regulates hepatic metabolic pathways to improve steatosis and inflammation. *Endocr Connect*. (2020) 9:755–68. 10.1530/EC-20-0152 32688339 PMC7424338

[95] Sui Y Liu Q Xu C Ganesan K Ye Z Li Y Non-alcoholic fatty liver disease promotes breast cancer progression through upregulated hepatic fibroblast growth factor 21. *Cell Death Dis*. (2024) 15:67. 10.1038/s41419-023-06386-8 38238320 PMC10796330

[96] Loomba R Sanyal A Kowdley K Bhatt D Alkhouri N Frias J Randomized, controlled trial of the FGF21 analogue pegozafermin in NASH. *N Engl J Med*. (2023) 389:998–1008. 10.1056/NEJMoa2304286 37356033 PMC10718287

[97] Noureddin M Rinella M Chalasani N Neff G Lucas K Rodriguez M Efruxifermin in compensated liver cirrhosis caused by MASH. *N Engl J Med*. (2025) 392:2413–24. 10.1056/NEJMoa2502242 40341827

[98] Xue Q Ji S Xu H Yu S. O- GlcNAcylation: a pro-survival response to acute stress in the cardiovascular and central nervous systems. *Eur J Med Res*. (2024) 29:174. 10.1186/s40001-024-01773-z 38491477 PMC10943874

[99] Guo Z Li H Qin W. [Precise identification of o-linked beta-n-acetylglucosamine peptides based on o-mesitylenesulfonylhydroxylamine elimination reaction]. *Se Pu.* (2021) 39:1182–90. 10.3724/SP.J.1123.2020.12024 34677013 PMC9404036

[100] Xiong W Lai X Lu J Li L Zhang J Duan X. O-GlcNAcylation orchestrates porcine oocyte maturation through maintaining mitochondrial dynamics and function. *Mol Hum Reprod*. (2024) 30:gaae003. 10.1093/molehr/gaae003 38265252

[101] Raab S Very N Duchêne B Rybarczyk P Jonckheere N El Yazidi-Belkoura I Evaluation of the expression of fatty acid synthase and O-GlcNAc transferase in patients with liver cancer by exploration of transcriptome databases and experimental approaches. *Oncol Lett*. (2022) 23:105. 10.3892/ol.2022.13225 35242233 PMC8848257

[102] Escobar E Seeley E Serrano-Negrón J Vocadlo D Brodbelt J. In situ imaging of O-linked β-N-acetylglucosamine using on-tissue hydrolysis and MALDI mass spectrometry. *Cancers*. (2023) 15:1224. 10.3390/cancers15041224 36831567 PMC9954453

[103] Zhou Y Li Z Xu M Zhang D Ling J Yu P O-GlycNacylation remission retards the progression of non-alcoholic fatty liver disease. *Cells*. (2022) 11:3637. 10.3390/cells11223637 36429065 PMC9688300

[104] Zhang Y Han S Li T Zhu L Wei F. Bisphenol A induces non-alcoholic fatty liver disease by promoting the O-GlcNAcylation of NLRP3. *Arch Physiol Biochem*. (2024) 130:814–22. 10.1080/13813455.2023.2288533 38038745

[105] Wu H Huang C Art J Liu H Hart G Zeltner NO-. GlcNAcylation is crucial for sympathetic neuron development, maintenance, functionality and contributes to peripheral neuropathy. *Front Neurosci*. (2023) 17:1137847. 10.3389/fnins.2023.1137847 37229433 PMC10203903

[106] Nakamoto A Ohashi N Sugawara L Morino K Ida S Perry R O-linked N-acetylglucosamine modification is essential for physiological adipose expansion induced by high-fat feeding. *Am J Physiol Endocrinol Metab*. (2023) 325:E46–61. 10.1152/ajpendo.00263.2022 37224467 PMC10292976

[107] Tang R Liu R Zha H Cheng Y Ling Z Li L. Gut microbiota induced epigenetic modifications in the non-alcoholic fatty liver disease pathogenesis. *Eng Life Sci*. (2024) 24:2300016. 10.1002/elsc.202300016 38708414 PMC11065334

[108] Zhang D Wang H Liu A Wang S Xu C Lan K The chronic consumption of dietary fructose promotes the gut Clostridium species imbalance and bile acid alterations in developing nonalcoholic fatty liver disease. *J Nutr Biochem*. (2023) 121:109434. 10.1016/j.jnutbio.2023.109434 37661068

[109] Han C Li Z Liu R Zhao Z Wang Y Zuo X Lonicerae flos polysaccharides improve nonalcoholic fatty liver disease by activating the adenosine 5’-monophosphate-activated protein kinase pathway and reshaping gut microbiota. *J Sci Food Agric*. (2023) 103:7721–38. 10.1002/jsfa.12854 37439182

[110] Song Q Zhang X. The role of gut-liver axis in gut microbiome dysbiosis associated NAFLD and NAFLD-HCC. *Biomedicines*. (2022) 10:524. 10.3390/biomedicines10030524 35327326 PMC8945287

[111] Kuraji R Shiba T Dong T Numabe Y Kapila Y. Periodontal treatment and microbiome-targeted therapy in management of periodontitis-related nonalcoholic fatty liver disease with oral and gut dysbiosis. *World J Gastroenterol*. (2023) 29:967–96. 10.3748/wjg.v29.i6.967 36844143 PMC9950865

[112] Mukhopadhya I Louis P. Gut microbiota-derived short-chain fatty acids and their role in human health and disease. *Nat Rev Microbiol*. (2025) 23:635–51. 10.1038/s41579-025-01183-w 40360779

[113] Tolhurst G Heffron H Lam Y Parker H Habib A Diakogiannaki E Short-chain fatty acids stimulate glucagon-like peptide-1 secretion via the G-protein-coupled receptor FFAR2. *Diabetes*. (2012) 61:364–71. 10.2337/db11-1019 22190648 PMC3266401

[114] Guo J Shi C Zhang Q Deng W Zhang L Chen Q Interventions for non-alcoholic liver disease: a gut microbial metabolites perspective. *Therap Adv Gastroenterol*. (2022) 15:17562848221138676. 10.1177/17562848221138676 36506748 PMC9730013

[115] Dmytriv T Storey K Lushchak V. Intestinal barrier permeability: the influence of gut microbiota, nutrition, and exercise. *Front Physiol*. (2024) 15:1380713. 10.3389/fphys.2024.1380713 39040079 PMC11260943

[116] Ohtani N Kamiya T Kawada N. Recent updates on the role of the gut-liver axis in the pathogenesis of NAFLD/NASH, HCC, and beyond. *Hepatol Commun*. (2023) 7:e0241. 10.1097/HC9.0000000000000241 37639702 PMC10462074

[117] Wang T Ishikawa T Sasaki M Chiba T. Oral and gut microbial dysbiosis and non-alcoholic fatty liver disease: the central role of Porphyromonas gingivalis. *Front Med*. (2022) 9:822190. 10.3389/fmed.2022.822190 35308549 PMC8924514

[118] Long C Zhou X Xia F Zhou B. Intestinal barrier dysfunction and gut microbiota in non-alcoholic fatty liver disease: assessment, mechanisms, and therapeutic considerations. *Biology*. (2024) 13:243. 10.3390/biology13040243 38666855 PMC11048184

[119] Álvarez-Mercado A Plaza-Diaz J. Dietary polysaccharides as modulators of the gut microbiota ecosystem: an update on their impact on health. *Nutrients*. (2022) 14:4116. 10.3390/nu14194116 36235768 PMC9573424

[120] Zhra M Elahi M Tariq A Abu-Zaid A Yaqinuddin A. Sirtuins and gut microbiota: dynamics in health and a journey from metabolic dysfunction to hepatocellular carcinoma. *Cells*. (2025) 14:466. 10.3390/cells14060466 40136715 PMC11941559

[121] Effenberger M Grander C Grabherr F Tilg H. Nonalcoholic fatty liver disease and the intestinal microbiome: an inseparable link. *J Clin Transl Hepatol*. (2023) 11:1498–507. 10.14218/JCTH.2023.00069 38161503 PMC10752805

[122] Yu T Luo L Xue J Tang W Wu X Yang F. Gut microbiota-NLRP3 inflammasome crosstalk in metabolic dysfunction-associated steatotic liver disease. *Clin Res Hepatol Gastroenterol*. (2024) 48:102458. 10.1016/j.clinre.2024.102458 39233138

[123] Han D Xu K Jin Z Xu Y Li Y Wang L Customized liver organoids as an advanced in vitro modeling and drug discovery platform for non-alcoholic fatty liver diseases. *Int J Biol Sci*. (2023) 19:3595–613. 10.7150/ijbs.85145 37497008 PMC10367556

[124] Zheng Z Zong Y Ma Y Tian Y Pang Y Zhang C Glucagon-like peptide-1 receptor: mechanisms and advances in therapy. *Signal Transduct Target Ther*. (2024) 9:234. 10.1038/s41392-024-01931-z 39289339 PMC11408715

[125] Amatya R Lee D Min K Shin M. Pharmaceutical strategies to improve druggability of potential drug candidates in nonalcoholic fatty liver disease therapy. *Pharmaceutics*. (2023) 15:1963. 10.3390/pharmaceutics15071963 37514148 PMC10386216

[126] Harrison S Bedossa P Guy C Schattenberg J Loomba R Taub R A phase 3, randomized, controlled trial of resmetirom in NASH with liver fibrosis. *N Engl J Med*. (2024) 390:497–509. 10.1056/NEJMoa2309000 38324483

[127] Copyright and License information. Phase 3 ESSENCE trial: semaglutide in metabolic dysfunction-associated steatohepatitis. *Gastroenterol Hepatol.* (2024) 12:6–7. PMC11784563 39896971

[128] Cooreman M Butler J Giugliano R Zannad F Dzen L Huot-Marchand P The pan-PPAR agonist lanifibranor improves cardiometabolic health in patients with metabolic dysfunction-associated steatohepatitis. *Nat Commun*. (2024) 15:3962. 10.1038/s41467-024-47919-9 38730247 PMC11087475

[129] Salomone F Sharaiha R Boškoski I. Endoscopic bariatric and metabolic therapies for non-alcoholic fatty liver disease: evidence and perspectives. *Liver Int*. (2020) 40:1262–8. 10.1111/liv.14441 32181573

[130] Chen Y Wang W Morgan M Robson T Annett S. Obesity, non-alcoholic fatty liver disease and hepatocellular carcinoma: current status and therapeutic targets. *Front Endocrinol*. (2023) 14:1148934. 10.3389/fendo.2023.1148934 37361533 PMC10286797

[131] Puri S Kirad S Muzaffar-Ur-Rehman M Mandal S Sharma P Sankaranarayanan M Lipogenic stearoyl-CoA desaturase-1 (SCD1) targeted virtual screening for chemical inhibitors: molecular docking / dynamics simulation and in vitro assessment of anti-NAFLD efficacy. *RSC Adv*. (2024) 14:31797–808. 10.1039/d4ra06037g 39380655 PMC11459445

[132] Tan D Ng C Lin S Pan X Tay P Lim W Clinical characteristics, surveillance, treatment allocation, and outcomes of non-alcoholic fatty liver disease-related hepatocellular carcinoma: a systematic review and meta-analysis. *Lancet Oncol*. (2022) 23:521–30. 10.1016/S1470-2045(22)00078-X 35255263 PMC9718369

[133] Li S Hao L Deng J Zhang J Hu X. Coptidis rhizoma and evodiae fructus against lipid droplet deposition in nonalcoholic fatty liver disease-related liver cancer by AKT. *Chem Biol Drug Des*. (2023) 102:828–42. 10.1111/cbdd.14295 37460115

[134] Mahdizadeh F Sobhi P Banaei S. A class of MicroRNAs as diagnostic biomarkers and therapeutic strategies in non-alcoholic fatty liver disease: a review. *Clin Res Hepatol Gastroenterol*. (2025) 49:102547. 10.1016/j.clinre.2025.102547 39924053

[135] Yang Y Zhou X Deng H Chen L Zhang X Wu S The role of O-GlcNAcylation in bone metabolic diseases. *Front Physiol*. (2024) 15:1416967. 10.3389/fphys.2024.1416967 38915778 PMC11194333

[136] Che W Zhao M Li X Li C Cho W Yu S. Current insights in molecular characterization of non-alcoholic fatty liver disease and treatment. *Front Endocrinol*. (2022) 13:1002916. 10.3389/fendo.2022.1002916 36523601 PMC9744925

[137] Zhu Z Chen Y Qin X Liu S Wang J Ren H. Multidimensional landscape of non-alcoholic fatty liver disease-related disease spectrum uncovered by big omics data: profiling evidence and new perspectives. *Smart Med*. (2023) 2:e20220029. 10.1002/SMMD.20220029 39188279 PMC11236021

[138] Fang J Pan L Gu Q Juengpanich S Zheng J Tong C Scientometric analysis of mTOR signaling pathway in liver disease. *Ann Transl Med*. (2020) 8:93. 10.21037/atm.2019.12.110 32175386 PMC7049061

[139] Zhao C Ji G Zhao X Ma T Li Y Wu W Single-cell data analysis reveals critical hepatic cells subpopulations in the progression of non-alcoholic fatty liver disease to non-alcoholic steatohepatitis. *Comb Chem High Throughput Screen*. (2025) 28:1251–63. 10.2174/0113862073303213240523095742 38803181

